# Mediterranean Dietary Pattern for Healthy and Active Aging: A Narrative Review of an Integrative and Sustainable Approach

**DOI:** 10.3390/nu16111725

**Published:** 2024-05-31

**Authors:** Polina Dobroslavska, Maria Leonor Silva, Filipa Vicente, Paula Pereira

**Affiliations:** Applied Nutrition Research Group (GENA), Nutrition Lab, Egas Moniz Center for Interdisciplinary Research (CiiEM), Egas Moniz School of Health & Science, Monte de Caparica, 2829-511 Almada, Portugal; pdobroslavska@gmail.com (P.D.); fvicente@egasmoniz.edu.pt (F.V.); pmpereira@egasmoniz.edu.pt (P.P.)

**Keywords:** Mediterranean diet, dietary pattern, active aging, hormetic, sustainable

## Abstract

The global population is on a trajectory of continuous growth, with estimates projecting an increase from 7.7 billion in 2019 to approximately 9.7 billion by 2050. Longevity is also expected to rise rapidly, with advancements in healthcare contributing to increased life expectancies and an increase in the maximum lifespan. The aging process is accompanied by different changes, often leading to a decline in daily life activities and an increased susceptibility to disease. Age-related changes can cause cellular damage and subsequent cellular death. Oxidative stress and inflammation play significant roles in this process contributing to molecular damage and mitochondrial dysfunction. Active aging has been associated with improved quality of life and a reduced risk of morbidity and premature mortality. In this context, the Mediterranean diet has emerged as a promising approach to promoting healthy aging and sustainability. The phytochemical compounds within the Mediterranean diet have been linked to a lower risk of developing cardiovascular disease, type 2 diabetes, obesity, cancer and neurodegenerative diseases. The findings of peer-reviewed articles regarding the use of the Mediterranean diet as a healthy and sustainable dietary pattern written in Portuguese, Spanish or English were included in this narrative literature review. This dietary pattern’s emphasis on the consumption of fresh and local food aligns with both health and environmental sustainability goals. This work provides a comprehensive review of the benefits of the Mediterranean diet and its components in a healthy aging process and highlights the importance of this dietary pattern as a sustainable approach.

## 1. Introduction

The global population is constantly growing; it is estimated that it could increase from 7.7 billion people worldwide in 2019 to 8.5 billion in 2030 and to around 9.7 billion in 2050. According to Vaupel, 2020, longevity will increase far more quickly than in the past and people’s life expectancy and maximum life span will also increase [1].

Even though global food supply has kept up with global population expansion, one in every eight people still lacks sufficient food, and many more consume low quality diets/consume too much [2]. Malnutrition and poor dietary habits are risk factors for non-communicable chronic diseases and mortality [3].

By 2050, the proportion of world’s population that will be 60 years old or older will nearly double from 12% to 22% [4]. In spite of the fact that the world’s population will live longer, the question of whether there will be a better quality of life remains.

Aging is related to biological, physiological, psychological and social changes [5,6] and has been associated with the lack of daily life activities and a increased susceptibility to disease [7]. The aging process results from different extrinsic events which lead to progressive cell damage and, consequently, cellular death [7,8]. Aging is linked to oxidative stress and inflammation status. Particularly, the generation of reactive oxygen species (ROS) and free radicals leads to molecular damage to the cells, organs and tissues [9] and, consequently, to mitochondrial dysfunction, inflammation processes and cells apoptosis [10,11]. Mitochondrial dysfunction seems to promote intracellular signaling impairment and membrane integrity disruption leading to apoptosis and cellular aging and to the dysregulation of metabolic pathways [10].

Active aging has shown a positive and consistent association with quality of life, particularly regarding physical activity, health and social services, social environment, economic, personal and behavior factors [12]. Also, active aging has been linked to a lower risk of morbidity and early death and improved chronic illness management [13]. In this context, strategies have been developed to promote healthy and active aging which have included nutrition and lifestyle modifications [10]. Beyond this, it is also important to implement those strategies sustainably by promoting sustainable healthy eating patterns, because people can live longer but there is no planet B [14].

There is robust evidence for the role of diet and dietary habits in health promotion and disease prevention. This had supported the establishment of dietary guidelines in several countries. However, the sustainability of these food choices is not always properly addressed. In fact, a previous review showed that food choices with lower greenhouse emissions would not result in improvements in terms of nutritional quality or health outcomes [15]. Considering the heterogeneity of food habits and food choices around the world, the use of recognized dietary patterns instead of specific food and food group recommendations can be a more consistent approach to establish what is a healthy diet and evaluate the possible influence of diet on health and sustainability outcomes [16].

Within the several recognized and established dietary patterns, it is possible to highlight the “Mediterranean diet”. In spite of the fact that it is commonly called the “Mediterranean diet” (MD), it is in fact a way of life that combines knowledge, practices, tradition and culture. Furthermore, the MD has been shown to exert a strong influence on promoting health, reducing mortality [17] and preventing age-related disease due to the phytochemical compounds within it [18]. The phytochemical compounds present in the Mediterranean diet seem to act as hormetins [18]. Hormesis, described as a positive or stimulating effect brought on by exposure to small amounts of a substance that is known to be harmful at larger concentrations, can contribute to the development of adaptive responses against cellular damage and can be used as an anti-aging strategy [19].

The Mediterranean diet has been also demonstrated to have a beneficial impact on the hallmarks of aging, decreasing the risk of age-related disease due to decreases in oxidative stress and inflammation [20].

In addition to health benefits and positive effects on aging, the Mediterranean diet has evolved into a dietary pattern in which food, culture, people, the environment and sustainability are integrated, being recognized not only as the healthiest food model but also the one with the lowest environmental footprint. The development of the Mediterranean diet concept contributes to the promotion of an integrative approach to healthy aging [21,22].

The aim of the present work is to provide a comprehensive review of the benefits of the Mediterranean diet and its components to a healthy aging process and highlight the importance of this dietary pattern as a sustainable approach to aging.

## 2. Search Methodology and Criteria

This narrative review used the results available from the literature regarding the use of the Mediterranean diet as an integrative approach towards a healthy and sustainable dietary pattern. The literature search was conducted from July 2023 up to March 2024 on the Medline, Cochrane Library and PubMed databases. The following terms were considered in this search: “Mediterranean diet”, “sustainable”, “one health”, “dietary pattern”, “hormesis”, “health aging” and “active aging”. Articles written in Portuguese, Spanish or English and published in the last 10 years were included in this literature review to gather the most recent data. The process of analyzing the articles we found was carried out in stages. Initially, the articles were assessed based on the title and in the abstract, and then the articles were analyzed based on full text. Specifically, only systematic reviews were considered to establish the benefits of MD in different health outcomes. This choice was due to the high level of evidence from this type of work and its importance in providing relevant and unbiased data [23,24].

## 3. The Mediterranean Diet as a Lifestyle

The Mediterranean diet has been studied by individuals in the scientific community, such as Ancel Keys, for 60 years [25]. The literature has shown that there is an inverse relationship between the Mediterranean diet and the incidence of pathologies such as cardiovascular disease, type 2 diabetes, obesity, cancer, metabolic syndrome and neurodegenerative diseases [26]. A diet that is close to the Mediterranean pattern in the long term is associated with health benefits and should be one of the main targets of public health strategies [27].

The Mediterranean diet is a sequence of various arts, techniques and patterns of life, in addition to an environment of intervention and reflection [26,28]. The principles of the Mediterranean pattern developed over 5000 years ago, influenced by different civilizations, making the Mediterranean diet a fusion of different food cultures present in the Mediterranean region [22]. The Mediterranean diet is a cultural model that has existed in the Mediterranean area for thousands of years where, for centuries, Greek, Roman, Phoenician, Arabic and other communities have shared cultural heritage, knowledge, traditions, genes, plants and animals, which has influenced the way they live. The Mediterranean diet was recognized as Intangible Cultural Heritage of Humanity (2010) by UNESCO and the food pyramid was subsequently presented to communicate this to the public, health professionals and stakeholders, with the aim of boosting adherence to this dietary pattern [29].

The Mediterranean diet is based on food, conviviality, food sustainable production and lifestyle [30]. This food pattern promotes the daily consumption of various fresh vegetables and fruit, nuts and seeds; the regular ingestion of whole grain products; the consumption of legumes several times per week; the use of extra virgin olive oil for cooking and for seasoning as the main source of fat; the utilization of herbs and spices for flavor instead of salt; the infrequent consumption of sweets cakes, and dairy desserts; the consumption of fish and seafood two to three times per week; the daily consumption of dairy products, in particular yogurt; the consumption of eggs two to four times per week; the infrequent consumption of red/processed meat; and ingesting water as the main beverage and drinking moderate amounts of wine, particularly red wine, with meals [29]. Furthermore, the Mediterranean diet promotes a connection with and respect for nature; flavorsome cooking; moderate physical activity every day; preparing and consuming meals in the company of other people; and having an appropriate amount of rest [26].

The Mediterranean diet stimulates and respects natural resources and seasonality. Promoting and encouraging adherence to the Mediterranean pattern, and incorporating a greater proportion of plant-based products as opposed to animal-based products, can play an important role in preserving the environment. Food choices can have a profound effect on human health and the environment. Less obvious is the impact on the health and welfare of animals and food producers. The Mediterranean food pattern and lifestyle stems from sustainable ecologic agricultural practices, resulting in tangible benefits in all three domains [31].

## 4. Mediterranean Diet, Non-Communicable Chronic Diseases and Mortality

Non-communicable diseases are one of the main causes of mortality and morbidity worldwide, and place a significant burden on global public health services. According to the World Health Organization (WHO), non-communicable diseases are responsible for most deaths and disability cases worldwide. Global data indicate that cardiovascular diseases are directly responsible for 17.9 million deaths per year, followed by cancer (9.3 million), chronic respiratory diseases (4.1 million) and diabetes (2.0 million) [24,25].

The Mediterranean dietary pattern had been associated not only with a better health status [32] but evidence of the effects of this dietary pattern in chronic disease prevention is constantly growing. As presented in Table 1, considering only the most recent years, there are a number of reliable systematic reviews showing its protective effect. A high adherence to the Mediterranean diet had been associated with a reduced risk of all-cause mortality, cardiovascular disease risk, type 2 diabetes, cancer risk and mortality from all causes in cancer survivors [17,33,34,35,36,37,38]. Table 1 show a summary of the systematic reviews related to the relationship between adherence to the Mediterranean diet and the risk of developing different non-communicable chronic diseases and cognitive decline with updated data from over the last few years and incorporating relevant new evidence.

Furthermore, cognitive decline and dementia are major comorbidities in the elderly. In fact, dementia is the seventh most common cause of death and the most common cause of illness in older adults. According to the WHO, there are currently more than 55 million confirmed cases of dementia worldwide, and the number of new cases is increasing at a rate of 10 million per year. In addition to this, the number of people with dementia is projected to grow to 78 million by 2030 and 139 million by 2050 [39].

The systematic review conducted by Fu and colleagues showed that high adherence to this dietary pattern was associated with a lower risk of mild cognitive impairment and Alzheimer’s disease [37]. Kheirouri et al.’s review suggested multiple possible components in Mediterranean diet foods that can have protective effects on the brain and nervous system, namely reducing oxidative stress, inflammation and the accumulation of AB plaques [38]. The effects of these compounds will be discussed in the following subsection.

## 5. An Overview of Hermetic and Health Effects of the Mediterranean Diet Food Components

The benefits of the Mediterranean diet can also be linked to a healthy aging process through the hormetic response [10,18,40]. The literature has shown that the phytochemical compounds within the Mediterranean diet, such as kaempferol, quercetin, capsaicin, resveratrol, curcumin, luteolin, fisetin, sesamin and resveratrol, can slow down the aging process, reduce susceptibility to chronic disease and thus increase longevity [41]. The aging process is caused by endogenous and exogenous cellular stressors that lead to DNA damage, mutations, dysfunctional protein accumulation (heat shock protein), oxidative stress, mitochondrial dysfunctional and inflammation [42]. The potential mechanism of action responsible for phytochemicals’ anti-aging benefits are related to the adaptative stress response pathways [18].

The phytochemical compounds in some foods that are present in the Mediterranean diet such as carotenoids, polyphenols, isoprenoids, phytosterols, saponins, polyunsaturated fatty acids (PUFA), dietary fiber and polysaccharides have been recognized for promoting healthy aging by acting as antioxidants and/or anti-inflammatories [43]. Additionally, micronutrients, namely vitamins (vitamin E, C, D and A), minerals and trace elements (selenium, copper and zinc), can also exert beneficial effects on health by inducing antioxidant enzymes [44]. The phytochemical compounds act as antioxidants which are involved in biological processes and defense through the inhibition of reactive oxygen species (ROS), such as superoxide anions, hydrogen peroxide, hydroxyl radicals, singlet oxygen and others. The ROS can affect the cellular membrane leading to the breakdown of peptide chains and lipid peroxidation [45]. Additionally, the resveratrol, catechin, quercetin and curcumin also seem to prevent aging through the induction of an autophagy cellular mechanism, which is promoted by mTOR [46]. The use of natural bioactive compounds at lower doses has been extensively studied regarding their potential mechanisms of action. Studies have reported that the anti-inflammatory activity of curcumin results from the promotion of damage repair through an enhanced heat shock protein response. Resveratrol stimulates cell proliferation via ERK1/2 activation and thus improves resilience to inflammatory stress. Also, the linoleic acid increases the NADAPH oxidases levels and consequently improves the hepatic redox status [47,48,49].

In addition to the hormetic effects of bioactive components and their antioxidant properties, other food components, such as epigallocatechin gallate, polysaccharides, polyphenols, fisetin and berberine, have been reported to have beneficial effects on health. The results of various clinical trials revealed that these bioactive compounds seem to be promising in improving type 2 diabetes mellitus prognosis, increasing insulin sensitivity, reducing cognitive impairment and improvements in cardiovascular, blood pressure control and cancer biomarkers [50,51,52,53,54].

Fruits and vegetables are rich in fiber, minerals, vitamins, polyphenolic compounds (such as, cinnamic acids, flavonoids, tannins) and carotenoids, which contribute to beneficial effects on health [55]. Studies suggest that the consumption of fruits and vegetables contributes to a lower risk of type 2 diabetes [56], prevents cardiovascular disease [57], improves mental health [58] and reduces all-cause mortality [59].

Legumes are another food component that is consumed more in a Mediterranean diet pattern and are rich in essential fatty acids, protein, fiber, phytosterols, vitamins and minerals [60]. According to clinical trials, the ingestion of legumes ingestion shows beneficial effects on glycemia control and glycated hemoglobin A1c [61]. The consumption of legumes has been also associated with a lower risk of cardiovascular disease [62]. Nuts are a source of monounsaturated (MUFAs) and polyunsaturated fatty acids (PUFAs) and phenols, flavonoids, isoflavonoids, minerals and fiber. A systematic review and meta-analysis of randomized controlled trials suggested that the consumption of pistachio nuts can improve the lipid profile, especially total cholesterol, low-density lipoprotein (LDL) and triglycerides [63].

Whole grains are also a source of polyphenols such as ferulic acid, oxalic acid, p-coumaric acid; as well as dietary fiber, minerals and vitamins [64]. In addition to these components, whole grains supply carbohydrate and protein [65]. Several studies have shown that the consumption of whole cereal grains (such as wheat, barley, oats and rice) as part of a healthy diet has a positive effect by reducing the risk of coronary heart disease, type 2 diabetes, obesity and cancer [64,65].

Fish, such as sardines, mackerel, tuna and others, are rich in omega-3 long chain PUFAs (eicosapentaenoic acid and docosahexaenoic acid), protein and minerals (such as iodine, selenium, zinc, magnesium and calcium) [66]. Fish consumption has been demonstrated to have a positive impact on health, especially by reducing the risk of cardiovascular disease and improving the lipid profile [67]. The consumption of fish and their bioactive compounds also decreases hepatotoxicity and hepatic injury and enhances antioxidant enzyme activities [68]. The Mediterranean dietary pattern is also an example of making sustainable and safe food choices with regard to fish; in addition to the advice on moderated consumption, most common fish and shellfish species (e.g., sardines, seabream, squid) have mercury concentrations below EU thresholds [69].

Olive oil is the main source of fat in the Mediterranean diet, specifically MUFAs which are are rich in polyphenols and vitamin E [70]. The consumption of extra virgin olive oil contributes to cardiovascular disease protection, a reduction in the LDL concentration and an increase high-density lipoprotein (HDL) concentrations. Additionally, olive oil seems to reduce pro-inflammatory markers (interleukin-6 and tumor necrosis factor) and stimulate beneficial gut bacteria [71]. Olive oil plays a fundamental role in oxidative stress regulation in healthy and non-healthy individuals due to its high phenolic content [72].

Red wine provides polyphenol compounds such as flavonoids (catechin, epicatechin, quercetin and anthocyanins), resveratrol and tannins. These bioactive compounds have been reported to have a beneficial effect on cardiovascular disease and obesity due to their antioxidant properties [73,74]. Several potential mechanisms have been attributed to explain the benefits of the Mediterranean diet food components, including lipid profile, glycemia control, endothelial functions, oxidative stress and inflammation [75].

## 6. Mediterranean Diet: Ensuring Healthier and Sustainable Food Choices through an Integrative Health Approach

The recent global nutritional transition is due to the widespread of the Western, urban and cultural economy, which are driven by food production and consumption, technology and the homogenization of eating behaviors [29,76]. There is an urgent need to launch a new strategy for the development of the concept of food sustainability and its use in the different contexts of industrialized and developing countries to guarantee food safety and quality [77]. The Mediterranean area is also undergoing a “Nutritional Transition”, in which malnutrition coexists with overweight, obesity and diet-related chronic diseases [24]. Regarding obesity, overweight and diet-related chronic diseases, the World Health Organization (WHO) published a report in 2022 on the current state of obesity in the European region, referring to strategies for the prevention and control of chronic diseases. Excess weight is one of the greatest risk factors for mortality and disability in the region, being responsible for more than 10% of all deaths in this region each year and accounting for 7% of all years lived with disability [78].

Changes to the current dietary pattern through the implementation of nutritional recommendations have also been associated with a reduction in the intake of calories, carbohydrates, sugar, total fat and saturated fat, which are related to significant weight loss, a reduction in waist circumference and an increase in high-density lipoprotein (HDL) and a decrease in total cholesterol and low-density lipoprotein (LDL) [79,80]. Long-term dietary changes are difficult when the desired goal is consistency; however, food education has the potential to optimize dietary behavior changes, promoting a greater adherence to a healthier dietary intake in the long term [80]. Low-cost foods are the most energy-rich (high in fat and sugar) and nutrient-poor, inducing both overweight and nutritional deficiencies due to the consequences of food choices determined by household income and levels of education [81]. In 2020, the European Parliament defined, through the “Farm to Fork Strategy”, a strategy for building a sustainable food system in the European Union, which aims to protect the environment and guarantee healthy food and farmers’ livelihoods. On the other hand, the FAO has proposed a framework for sustainable food systems, which addresses current and future needs, including reducing poverty and improving food security and nutrition [78]. Sustainable food systems bring benefits to all citizens, but require adherence to a healthy diet, strategies to reduce food losses and waste and an increase in food production from both agriculture and industry. Policies aimed at achieving sustainable food systems must follow a multidimensional, gender-sensitive approach, and integrate all stages including transport, storage, processing, distribution, marketing and food consumption, considering all cultural aspects and ensuring fair trade at regional, national and international levels. Food and nutrition security can only be achieved if the entire population has access to nutritious food and is given the information and freedom they need to make appropriate nutritional choices [82]. These food systems should be based on the agro-ecological production of staple foods, including limited food animal husbandry, short-distance production–consumption networks, food processing and refinement, emphasizing important culinary skills, food and nutrition education, and firm links to positive cultural and local traits, as well as the appropriate use of recent technological tools. Improving biodiversity could be considered a strategy for sustainable food production and consumption [83].

To assess the Mediterranean pattern as a sustainable dietary pattern, the following indicators must be considered: the environment, nutrition, the economy and socio-cultural factors [84]. The use of sustainability indicators is essential for an integrated assessment of food standards [85]. For each of the dimensions of a sustainable food system there is a set of complementary indicators; however, scientific evidence shows that there is no uniformity in the way the sustainability of the Mediterranean diet can be assessed. The most used indicators for evaluating the pattern as sustainable are greenhouse gases, water use and agriculture. The nutritional and socio-cultural dimensions do not yet present representative data, but despite all the gaps in the scientific evidence, the Mediterranean diet has been reported as the most sustainable dietary pattern [76,84]. One of the main barriers considered to limit adherence to the Mediterranean diet is the ability of consumers at the time of purchase to consciously assess whether the food is healthy while respecting the environment and ensuring social sustainability [86]. Other barriers included demographic, motivational, hedonic and sensorial factors, such as, lower education levels [87], a lack of nutrition education [88], resistance to food habits change [89], low abilities to adhere to diets [90], low sensory appeal [91] and low motivation to adopt healthy foods cooking methods [90]. According to different studies, the socio-cultural issue also contributes to those barriers, including negative family influences [92], cultural differences [93] and stressors [93].

One of the objectives of the European Commission’s “From Plate to Plate” strategy is the creation of sustainable labelling (Med Index) that is capable of including the sustainability dimension, which covers the nutritional, climatic, environmental and social aspects of food products [94]. The creation of sustainable labelling aims to promote adherence to the Mediterranean diet, or foods that can be found anywhere in the world but contain the same beneficial characteristics as foods from the Mediterranean region. The Med Index cuts across all three pillars of sustainability, including nutritional, environmental and social aspects [94].

Nelson et al. (2020) conducted a systematic assessment of dietary patterns at the population level and revealed that the DASH, plant-based diets (like vegetarianism), Mediterranean diets and other diets strongly correspond with fewer environmental effects (such fewer greenhouse gas emissions) [95]. There is more and more literature supporting the preservation of the Mediterranean diet, traditions, land and biodiversity and highlighting its benefits in preventing serious non-communicable diseases [96]. However, adherence to the Mediterranean pattern is still a challenge as we can see from the results of the systematic review by Obeid et al. [97].

As previously discussed, the Mediterranean diet promotes frugality, prefers local and traditional foods and dishes, includes nutritious food and promotes health. In this context, all these principles are included in the FAO guidelines that define what a sustainable and healthy dietary pattern would be across the world. The Planeterranean diet is an attempt to get each country to rediscover its own heritage and develop healthier eating patterns based on traditional and local foods [98]. Current eating habits, which include diets of poor quality and variety, with the majority of energy intake coming from ultra-processed and high-glycemic foods, are one of the main causes of the global epidemic of obesity, metabolic and cardiovascular diseases. It is possible to identify specific fruits, vegetables, legumes, whole grains and sources of unsaturated fats that have nutritional contents and characteristics like those provided by foods typical of the Mediterranean pattern [99,100].

## 7. Limitations

In spite of the fact that research on nutrition science is constantly growing, most evidence is based on epidemiologic data and observational studies. The vast complexity of food habits and the impossibility of blinding participants in clinical trials make it almost impossible to overcome this challenging limitation. Despite the unbiased and consistent methodology used in this research, the present work is a narrative review, and therefore it offers a view of the state of the art of a specific subject but does not offer a sufficient level of evidence for decision making in terms of clinical practice.

## 8. Conclusions

This narrative review provides evidence that supports several of the positive aspects of adhering to the Mediterranean diet, suggesting that it benefits the health of both humans and the planet. The Mediterranean diet emerges as an essential factor in promoting active aging, offering numerous health benefits due to its bioactive compounds. The phytochemical components of the Mediterranean diet act as hormetic agents, stimulating adaptive responses to cellular damage and aging, reducing the risk of age-related diseases by mitigating oxidative stress and inflammation.

A healthy diet should promote both human and planet health. Sustainability and sustainable diets are now common trends in nutrition research. While food technology research continues to search for emergent solutions in the XXI century, an ancient dietary pattern could solve this challenge. Beyond recognized health benefits, the Mediterranean diet fosters a holistic lifestyle, integrating food, culture, the environment and sustainability. Rooted in ancient traditions, this dietary pattern promotes the consumption of fresh produce, nuts, olive oil and fish, while limiting the consumption of meat and processed foods. Its emphasis on natural resources, seasonality and respect for the environment aligns with the principles of sustainability. Therefore, health professionals, politicians and others should address and reinforce their efforts to improve public literacy and increase the adherence to this “glocally” beneficial dietary pattern to promote active and healthy aging.

In conclusion, the Mediterranean diet not only promotes active aging and prevents NCDs but also serves as a model for sustainable food choices. Embracing its principles can lead to healthier individuals and a more sustainable planet, highlighting the importance of integrating dietary patterns into broader environmental and societal considerations.

## Figures and Tables

**Table 1:** A summary results of systematic reviews about the relationship between Mediterranean diet adherence and the risk of developing different non-communicable chronic diseases and cognitive decline.

Reference	Outcome	Results
[17]	Adherence to the Mediterranean diet and primary and secondary prevention of cardiovascular disease	Higher adherence to the Mediterranean diet is associated with a reduced risk of all-cause mortality, cardiovascular disease, including myocardial infarction, stroke and cardiovascular mortality
[33]	Adherence to the Mediterranean diet and the risk of type 2 diabetes	Individuals with moderate to high adherence to the MD were less likely to develop diabetes
[34]	Adherence to the Mediterranean diet and cancer risk	Highest adherence to the Med. diet was related to a lower risk of cancer mortality in the general population, and all-cause mortality among cancer survivors as well as colorectal, head and neck, respiratory, gastric, liver and bladder cancer risks.
[35]	Diet and cancer risk (breast and colon)	Adherence to the Mediterranean diet emerged as a protective factor for colorectal and breast cancer.
[36]	Adherence to the Mediterranean diet and dementia and Alzheimer disease risk	Despite the relatively low risk reduction associated with higher adherence to Med. diet among the elderly, and it should be considered that this population is the most affected.
[37]	Adherence to the Mediterranean diet and cognitive health among healthy adults	Adherence to the Med. diet may reduce the risk of mild cognitive Impairment and AD
[38]	Adherence to the Mediterranean diet and cognitive performance	Adherence to the MIND diet may possibly be associated with an improved cognitive function in older adults. The MIND diet may be superior to other plant-rich diets for improving cognition
